# Puberty and oestral cycle length in captive female jaguars *Panthera onca*

**DOI:** 10.1093/conphys/coaa052

**Published:** 2020-06-16

**Authors:** Priscila Viau, Débora Cattaruzzi Rodini, Gisela Sobral, Gabriela Siqueira Martins, Ronaldo Gonçalves Morato, Claudio Alvarenga de Oliveira

**Affiliations:** 1 Departamento de Reprodução Animal, Faculdade de Medicina Veterinária e Zootecnia, Universidade de São Paulo, Av. Prof. Dr. Orlando Marques de Paiva, 87 Cidade Universitária Armando de Salles Oliveira, Butantã, 05508270, São Paulo (SP), Brazil; 2 Centro Nacional de Pesquisa e Conservação de Mamíferos Carnívoros, Instituto Chico Mendes de Conservação da Biodiversidade, Estrada Municipal Hisaichi Takebayashi, 8600, Bairro da Usina, Atibaia, 12952011 São Paulo, Brazil

**Keywords:** Felids, maturity, non-invasive, oestrogens, progestins, reproduction

## Abstract

Habitat loss and fragmentation have been leading jaguars to constant conflicts with humans, and as a result, jaguar populations have been declining over the last decades. Captive breeding is often a tool for species conservation, and it is not different for jaguars. However, success is far from optimal due to the lack of basic knowledge about species’ reproductive biology. In the present study, we assessed gonadal hormonal profiles of natural oestral cycles and puberty and compared our data to those of other wild felids. We collected faecal samples from two to seven times per week for 18 months from two adults and three pre-pubertal females. We defined baseline levels for progestins and oestrogens in order to estimate oestrous cycle length and age at puberty. We compared our data with 16 other species through generalized linear model, using weight and genus as two explanatory variables. Cycle length was 38.28 ± 2.52 days, ranging from 25 to 44 days, while sexual maturity was attained within 22 months. Due to our analysis of both hormonal and behavioural data, there is a variation between this research from other studies that employed only behavioural observations. Such difference may be caused by the absence of behavioural oestrous at the peri-pubertal period. When compared to wild felids of similar size, puberty and oestral cycle durations of the jaguar females fell within the same range. Our modelling showed that age at maturity was influenced mostly by size and only *Leopardus* partially explained the observed variation. Conversely, oestral cycle length did not differ among genera or size categories. Our study adds to the body literature in the reproductive endocrinology of wild felids, and because female gametes are more challenging to collect and preserve, a strong understanding on the female reproductive physiology is essential to assisted reproduction and wild population viability assessment.

## Introduction

Felids are considered hypercarnivores, which are animals that present morphological specializations for a diet composed almost exclusively of flesh. Big cats generally require substantial amounts of food and also large home ranges (Loveridge *et al*., 2010). Species with these characteristics are often considered as top predators, regulating prey population and providing essential ecosystem services (Treves & Karanth, 2003). However, deforestation often leads to increased contact with humans, converging to negative interactions and, together with illegal poaching, there is a worldwide trend in felid population decline (Wildt *et al*., 1979; Lasley *et al*., 1994; Loveridge *et al*., 2010; Quigley *et al*., 2018).

The most successful conservation strategies acknowledge the importance of reproductive physiology parameters (Cooke *et al*., 2013). Understanding the reproductive processes under natural conditions (*in situ* populations) provides a guideline for *ex situ* conservation efforts. However, captive populations provide a more refined assessment of reproductive parameters and, consequently, the development of assisted reproductive techniques. Such knowledge is vital for the maintenance of viable and healthy wild populations (Cooke *et al*., 2010).

The success in applying assisted reproductive technologies is <10% in most felids (Brown *et al*., 1996). Much of it is due to the lack of knowledge about fundamental aspects of their reproductive biology so that we are unable to mimic or recognize natural requirements. In this sense, ovarian steroid profiles are still unknown for half of the felid species (Brown, 2011). The ovarian activity has two main aspects: when the activity begins (puberty), and the oestrus cycle length. For most of the felid species studied so far, age at maturity is of 1 to 2 years (Schmidt *et al*., 1979, 1988; Bonney *et al*., 1981; Santiapillai *et al*., 1982; Bernard and Stuart, 1987; Graham *et al*., 1995; Eisenberg and Redford, 1999; Brown, 2011; Barnes *et al*., 2016), except for the tiger *Panthera tigris* (Nowell and Jackson, 1996; Graham *et al*., 2006) that reaches maturity around 4 years of age.

As for oestral cycle duration, it varies across genera, being as short as 7 days in the cheetah, *Acinonyx jubatus* (Graham *et al*., 1995; Brown, 2011), and as long as 56 days in the lioness, *Panthera leo* (Schmidt *et al*., 1979; Graham *et al*., 1995). There are also differences regarding methods, and behavioural data usually provides a lengthier oestral cycle than studies based on hormonal profiles (e.g. Tewes *et al*., 1987).

The jaguar (*Panthera onca*) is the largest South American cat, and although charismatic, it is considered as the carnivore least compatible with anthropic reality (Cavalcanti *et al*., 2010). Overall, there is a decreasing trend in this species’ populations in several countries (de la Torre *et al*., 2017), with a boost in captive breeding programs. Wildt *et al*. (1979) conducted one of the earliest studies on the reproductive physiology of jaguars 40 years ago. However, ovulation in the referred females was artificially induced, and few studies to date have tried to assess naturally occurring oestral cycle, and none have addressed puberty (e.g. Barnes *et al*., 2016).

Therefore, the present study provides, for the first time, the hormonal profiles of naturally occurring oestral cycles, aiming to contribute to the knowledge on ovarian function and sexual maturation in female jaguars. We hypothesize that sexual maturity will fall within the known range described for felids (1–2 years old). Considering oestral cycle length, we believe that its duration will be shorter compared to studies reporting behavioural oestrus. Since it varied greatly, we predict that the observed range will either be closer to its sister-species (21–56 days, mean 33) or comparable with cats of similar size (7–56 days, mean 29).

## Material and methods

### Humane care guidelines

The present research agrees with Ethical Principals in Animal Research adopted by the Bioethics Commission of the School of Veterinary Medicine and Animal Science of the University of São Paulo under the protocol number 1425/2008.

### Subjects

We assessed five female jaguars, two adults (AA01 and AA02) and three pre-pubertal individuals (PP01, PP02 and PP03). Of the three pre-pubertal cats, two were sisters (PP01 and PP02). Adult females and PP03 were housed individually, while the sisters were housed together. However, the sisters slept in separate cages, allowing sample collection in the following morning without cross contamination. The estimated age, stimulatory cues (direct or indirect) received from conspecific, origin and holding institution are shown in Table 1.

**Table 1:** List of the animals’ identification, their estimated age, exteroceptive cues available to females (indirect—only visual, olfactory and auditory stimuli; direct—all plus tactile stimulus), their origin and current captive colony location

**Animal**	**Age**	**Exteroceptive cues**	**Captive colony**
AA01	>24 months	Indirect with unrelated males	FPZSP^1^
AA02	>24 months	Indirect with unrelated males	RioZoo^2^
PP01	11 months	Direct with PP brother	PZMQB^3^
PP02	11 months	Direct with PP brother	PZMQB^3^
PP03	15 months	Indirect with PP brother	CCFS^4^

Age was estimated according to Cavalcanti and Gese (2010)

^1^Fundação Parque Zoológico de São Paulo (São Paulo, Brazil); ^2^Fundação RioZoo (Rio de Janeiro, Brazil); ^3^Parque Zoológico Municipal Quinzinho de Barros (Sorocaba, Brazil); ^4^Centro de Conservação da Fauna Silvestre Ilha Solteira (São Paulo, Brazil)

All animals were exposed to natural climatic conditions (photoperiod, temperature, etc.). Water was provided *ad libitum*, and the diet was offered in the afternoon according to the institution’s routine.

### Sample collection and RIA analysis

We collected 953 faecal samples from all females two to seven times per week for 18 months (from January 1998 to August 1999). Because there were periods of intermittence, some of the cycles detected were incomplete and, therefore, precise cycle length was not always possible.

Only fresh samples and those that were not cross-contaminated between individuals were collected and stored at −20°C until hormonal extraction. Extraction of faecal oestrogen and progestin metabolites was performed using the protocol previously reported by Brown *et al*. (1994). Briefly, we added 90% ethanol to 0.2 g of dry faeces, which was incubated at 90°C for 25 min after boiling, refilling the evaporated ethanol to initial volume. Samples were homogenized and centrifuged for 15 min, with the supernatant transferred to a new test tube. The remaining pellet was resuspended in 5 mL 90% ethanol and centrifuged, with the two supernatants combined in the same test tube. The tubes were dried and reconstituted with 1 mL of 100% methanol, followed by an ultrasonic bath for 15 min, and then stored at −20°C until analysis.

Analyses of gonadal hormone metabolites were performed using radioimmunoassay utilizing a commercial kit (Coat A Count, Diagnostic Products Corporation, Los Angeles, CA, USA) that had been previously validated for the jaguar (Viau *et al*., 2005). Because it was published in Portuguese, we will provide the basic values necessary for validation. Serial dilutions of pooled faecal extracts paralleled to the standard curve (*r* = 0.96, *P* < 0.001 for progestins, and *r* = 0.97, *P* < 0.001 for oestrogens). Intra- and inter-assay coefficient variations were <13% and <2.1%, respectively. Assay sensitivity was 1.47 pg/mL for oestrogens and 0.005 ng/mL for progestins.

### Steroid stability over time

In order to assess whether progesterone metabolites would remain stable over time and to provide a safer, cheaper and eco-friendlier technique, we selected a subset of 20 samples and conducted an enzyme immunoassay (EIA) after 8 years of storage. According to Kumar and Umapathy (2019), RIA employs radioactive material and waste and costly equipment.

For EIA, extraction followed and Illera *et al*. (2003). The assay used progesterone antibody CL425 and progesterone-HRP conjugate (Coralie Munro, UC Davis, California), and we also validated the assay through parallelism (*r* = 0.98, *P* < 0.001). The assay sensitivity was 0.14 pg/well, and all coefficients of variation were below 10%.

### Oestral cycle length

We considered peaks when oestrogen values exceed the mean ± 1.5 SD the baseline for three consecutive days. We defined the length of the oestral cycle based on the time between two consecutive oestrogen peaks. As stated previously, the interment sampling routine did not allow for precise cycle duration and, thus, we only included cycles whenever they were complete.

To facilitate visualization, we aligned cycles assigning the first day that we detected baseline oestrogen levels as day zero (D0) and the preceding period as oestrus phase. Conversely, the following days were considered as inter-oestrus.

### Basal levels and oestral cycle definition

We defined baseline values of oestrogen by applying successive mean recalculations on only adult females. Whenever the values of the faecal metabolites exceeded the mean **±** 1.5 SD, we excluded those values (Brown *et al*., 1996) and calculated new means. After all the recalculations, the oestrogen baseline value was 31.06 ± 1.3 ng/g dry faces for AA01 and 24.35 ± 2.68 ng/g dry faces for AA02. For progestin evaluation, we adopted the same procedure, although considering the mean ± 2.0 SD (Brown *et al*., 1996). Progestin baseline levels were 0.10 ± 0.003 μg/g dry faeces for AA01 and 0.19 ± 0.020 μg/g dry faeces for AA02. Because progestin values oscillated around the basal values, we adopted oestrogen baseline levels as threshold to define oestrus and interoestrus. As most measurements from pre-pubertal females remained at the baseline level, the onset of ovarian activity was detected once oestrogen levels surpassed the mean ± 1.5 SD (Brown *et al*., 1996).

### Statistical analysis

We analysed data using the SAS System for Windows software (SAS Institute Inc, Cary, NC, USA, 2000) and R (2019). Variables were tested for normality by Kolmogorov–Smirnov, and we employed Bartlett test for homogeneity of variances. Our data did not meet the premises for parametric tests, so we used Wilcoxon tests to compare groups (Shille *et al*., 1983). All data referring to the hormonal concentrations were expressed as means ± standard error of the mean.

In order to compare with other felids, we gathered available information on the same reproductive parameters (age at maturity—AgM—and oestral cycle length—ECL). Only 10 genera had available information on these variables, a reduced subset for proper statistical analysis. Regardless, this dataset and its residuals were normally distributed, allowing the use of Pearson’s correlation to understand if weight and maturity, and weight and oestral cycle length, were correlated. We also employed one-way ANOVA and Tukey test *post hoc* to separate AgM and ECL mean values among groups. ‘Groups’ were genus and weight category (small <6.5 kg, medium up to 20 kg and large above 20 kg, following Nowell and Jackson, 1996). Finally, we tested if AgM and ECL variation was explained by size category and genus with linear regression. Because some genera were underrepresented, we excluded those with less than three studies, namely *Caracal*, *Lynx*, *Otocolobus* and *Prionailurus*, from genus comparison, but not from size comparison. However, it is important to emphasize that this approach does not consider that some genera may be more closely related than others. We could not assess the interaction of weight and genus due to our reduced sample size.

## Results

### Adult females

**Figure 1:**
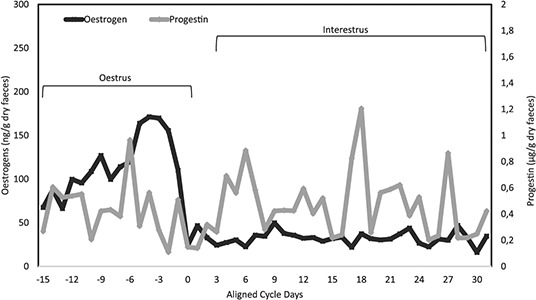
Mean values of faecal oestrogen and progestins from seven oestral cycles of adult female jaguars (*Panthera onca*, *n* = 2), discriminating oestrus and inter-oestrus phases (D0 = first basal value of faecal oestrogens).

Based on seven oestral cycles from the two adult females, cycle length was 38.28 ± 2.52 days, ranging from 25 to 44 days (Fig. 1). From a single animal, five complete cycles (AA01) were detected, and the cycle length was 36.20 ± 3.09 days, ranging from 25 to 41 days. As for the AA02, two complete oestral cycles were detected, with a duration of 43.50 ± 0.5 days.

The oestrus phase had an average duration of 10.42  ±  1.15days (ranging from 7 to 15), and the inter-oestrus phase lasted for ~28 ± 1.43 days (ranging from 28 to 31 days). The values for each adult female are provided in Table 2, while the mean values for adult and pre-pubertal females are included in Table 3.

**Table 2:** Basal and peak values of oestrogen (ng/g dry faeces) and progestins (μg/g dry faeces) for each adult female

	**Oestrogen (ng/g dry faeces)**		**Progestin (μg/g dry faeces)**
**Individual**	**Basal**	**Peak**	**Basal**
AA01 (*n* = 380)	31.06 (±1.3)	120.34 (±11.82)	0.44 (±0.25)
AA02 (*n* = 129)	24.35 (±2.68)	174.13 (±18.70)	1.65 (±0.31)

**Table 3:** Mean and range values of oestrogen (ng/g dry faeces) and progestins (μg/g dry faeces) for each group

	**Oestrogen (ng/g dry faeces)**		**Progestin (μg/g dry faeces)**	
**Group**	**Mean**	**Range**	**Mean**	**Range**
Adults (n = 509)	68.99 (±6.55)	3.50–609.37	0.85 (±0.09)	0.08–6.51
Pre-pubertals (n = 262)	10.97 (±0.91)	0.28–59.16	0.26 (±0.04)	0.02–4.44

### Pre-pubertal females

**Figure 2:**
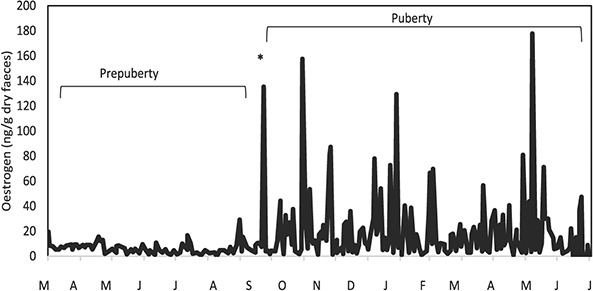
Longitudinal profile of faecal oestrogens for a pre-pubertal female (PP03) jaguar (*Panthera onca*). Asterisk refers to the onset of ovarian activity.

**Figure 3:**
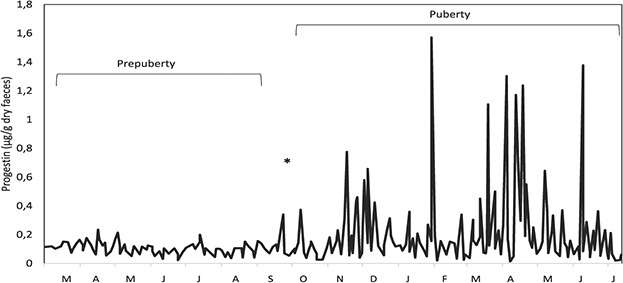
Longitudinal profile of faecal progestins for a pre-pubertal female (PP03) jaguar (*Panthera onca*). Asterisk refers to the onset of ovarian activity.

The female PP03 was considered as a model for the other pre-pubescent females (PP01 and PP02) since it was possible to clearly detect the differences between pre-pubertal and pubertal periods (Figs 2 and 3; Table 4). All animals entered the peri-pubertal phase with an approximate age of 20 months (18–20 months of age).

**Table 4:** Mean and range values of oestrogen (ng/g dry faeces) and progestins (μg/g dry faeces) for each maturation stage from female PP03

	**Oestrogen (ng/g dry faeces)**		**Progestin (μg/g dry faeces)**	
**Maturation stage**	**Mean**	**Range**	**Mean**	**Range**
Pre-pubescent (*n* = 109)	6.11 (±0.41)	0.28–29.99	0.10 (±0.003)	0.02–0.23
Pubescent (*n* = 165)	20.21 (±2.15)^*^	0.80–177.09	0.19 (±0.020)^*^	0.01–1.56

^*^ *P* < 0.05 (statistical difference between pre-pubertal and pubertal stages).

Wilcoxon rank-test showed that there was a significant difference between pre-pubertal and adult females concerning both progestin and oestrogen levels. However, assessing pre-pubertal females only, oestrogen levels differed from pre-puberty and puberty phases, but not progestins.

### Steroid stability: RIA and EIA comparison

Comparing RIA results obtained from samples assayed 8 years apart, both results showed a significant positive correlation (*r* = 0.98; *P* < 0.001). All the patterns (oestral cycle duration, puberty, as well as progestin and oestrogen profiles) demonstrated above through RIA were recovered using EIA.

### Comparison with other felids

Our survey showed that there is reproductive information for only 10 felid genera (Table 5). There was a strong correlation between AgM and size (*P* < 0.005) and between ECL and size (*P* < 0.04). Our GLM showed that only *Leopardus* genus had a positive influence on AgM (*P* < 0.05). However, considering ECL, there was no effect either from genera or size. Finally, one-way ANOVA and Tukey *post hoc* test detected differences in AgM among size categories (large differentiates from both medium and small sized cats). Only two genera differed in AgM, namely *Leopardus* and *Panthera*. Conversely, ECL were statistically equal among genus and size categories. Hence, AgM is mostly influenced by weight and genus, while ECL is fairly constant among taxa and sizes.

**Table 5:** List of species, its respective size and size category (based on Nowell and Jackson, 1996), oestral cycle length (ECL), age at maturity (AgM) and the references where the information is available

**Species**	**Size (kg)**	**Size category**	**ECL (days)**	**AgM (years)**	**References**
*Panthera tigris*	136	Large	29	3–4 years	a; b; c; d; e
*Panthera leo*	126	Large	38.5	2 years	a; b; f; g
*Panthera onca*	56	Large	37.5	2 years	a; h; this study
*Puma concolor*	41	Large	16	2 years	a; e; i; j
*Acinonyx jubatus*	43	Large	14	2–3 years	a; b; k; l
*Panthera pardus*	40	Large	24.5	2.5 years	a; e; f; m; n; o; p
*Panthera uncia*	37.5	Large	32.5	2–3 years	a; b; e; q
*Lynx* sp*.*	15	Medium	NA	1–2 years	a; e; l
*Neofelis nebulosa*	20	Medium	27.5	2 years	a; e; l; r
*Caracal caracal*	10	Medium	23.5	0.5–1.5 years	a; b; s
*Leopardus pardalis*	9.0	Medium	18.4	1 years	a; t
*Prionailurus viverrinus*	6.8	Medium	20	2 years	u; v; w
*Leopardus tigrinus*	2.0	Small	16.7	2 years	a; t
*Leopardus wiedii*	3.2	Small	17.6	0.5 years	a; t
*Otocolobus manul*	3	Small	14	1 years	a; x
*Felis margarita*	3	Small	11.1	1 years	y
*Felis nigripes*	3.5	Small	11.9	1 years	y

**a** Nowell and Jackson, 1996; **b**Graham *et al*., 1995; **c**Graham *et al*., 2006; **d**Seal *et al*., 1985; **e**Brown and Comizzoli, 2018; **f**Schmidt *et al*., 1979; **g**Putman *et al*., 2010; **h**Barnes *et al*., 2016; **i**Bonney *et al*., 1981; **j**Eisenberg and Redford, 1999; **k**Brown *et al*., 1996; **l**Brown, 2011; **m**Sadleir, 1996; **n**de Haas van Dorsser *et al*., 2007; **o**Owen *et al*., 2010; **p**Cunningham and Gross**,** 2000; **q**Schmidt *et al*., 1993; **r**Yamada and Durrant, 1989; **s**Bernard and Stuart, 1987; **t**Moreira *et al*., 2001; **u**Santymire *et al*., 2011;**v**Kinzer & Groome, 2011; **w**Fazio *et al.*, 2016; **x**Brown *et al*., 2002; **y**Herrick *et al*., 2010.

## Discussion

### Oestral cycle length and oestrus duration

The cycle duration of seven natural cycles from two adult jaguars was 38.28 ± 2.52 days. This duration differs slightly from other studies. For instance, Wildt *et al*. (1979) estimated an oestral cycle of 47.2 ± 5.4 days. The referred study assessed only one female, associating behavioural observations with artificially induced ovulation protocols. As for studies based solely on behavioural observations, the cycle was longer, which lasted up to 65 days (Wildt *et al*., 1979; Tewes *et al*., 1987; Sadleir, 1996). By assessing oestrus duration, the work of Barnes *et al*. (2016), provided data for seven adult females in different reproductive situations. The females considered as a control in the referred study resembled the husbandry protocol applied in our study. The duration of oestrus found in our study was 10.42 ± 1.15 days, while those females were of 5.5 ± 0.4 days. Similarly, based on behavioural observations of four natural reproductive cycles, oestrous duration was 8 to 10 days (Jorge-Neto *et al*., 2010). It is important to emphasize that unbalance in either behaviour or hormonal regulation may lead to reproductive failure, and therefore, both need to be synchronized and working adequately.

In our study, adult females were stimulated only indirectly by males (without physical contact). It seems that this situation did not suppress follicular growth since there was oestrogen fluctuation (Shille *et al*., 1979). Nonetheless, due to the fluctuation pattern of progestins, it was not possible to detect ovulation, although it might have occurred. Traditionally, jaguars, like many other felids, are characterized as induced ovulators, which is when ovulation is induced by copulation (Wildt *et al*., 2010). However, there is evidence that other exteroceptive stimuli (tactile, visual, olfactory or auditory) may trigger ovulation (Wildt *et al*., 1979; Sadleir, 1996; Rekwot *et al*., 2001; Jorge-Neto *et al*., 2020). Non-mating ovulations were recorded in females housed together in other species (Bristol-Gould and Woodruff, 2006; Brown, 2011). Hence, ovulatory stimulation in felids is variable not only across species but also at an individual level.

### Puberty

We have found that females entered the peri-pubertal phase with an approximate age of 20 months (18–20 months of age), a length similar to those reported in the literature (Wildt *et al*., 1979). Based only on behavioural patterns, age at puberty estimation fell between 24 and 30 months (Wildt *et al*., 1979; Mondolfi and Hoogesteijn, 1986). The female PP03 was followed for 31 months, and her cycling was not like the adult females. Therefore, even after initiating ovarian cycling at 20 months, females may show irregular cycles for an extended period. In caracals, although the onset of the ovarian activity happened between 7–10 months, reproductive success was only achieved at the age of 14 months (Bernard and Stuart, 1987).

It is not uncommon that the hypothalamic–pituitary–gonadal axis is activated prior the expression of reproductive behaviour. Puberty is a process that, afterwards, an individual attains sexual maturity. In the review of Schultz and Sisk (2006), they have shown that individuals experimentally treated with steroid hormones did not express behaviour during pre-puberty. They concluded that the brain was not yet primed for reproductive behaviour activation.

### Comparison with other wild felids

Puberty (or maturity) is reasonably stable across Felidae (1–2 years old), with smaller species attaining sexual maturity at an earlier age (1 years old) than larger species (2 years old) (Schmidt *et al*., 1979; Bonney *et al*., 1981; Santiapillai *et al*., 1982; Bernard and Stuart, 1987; Graham *et al*., 1995; Brown, 2011). Some exceptions are, for instance, *Prionailurus*, an Asian wild cat of 6.8 kg (medium-sized) that attains maturity around 2 years of age (Kinzer & Groome, 2011; Fazio et al. 2016; Santymire *et al*. 2011). In our analysis, size explained a large portion of the differences observed in maturity, while the only genus that influenced variation was *Leopardus*.

In domestic cats, the onset of puberty varies according to breed and body weight (Root *et al*., 1995; Johnston *et al*., 2001). This trend is not restricted to felids but is ubiquitous to mammals. For instance, smaller mammalian species tend to attain maturity at relatively smaller sizes than larger mammals (McClintock, 1983; Plotka *et al*., 1988). However, it is impressive that a smaller magnitude of just 20 kg is enough to explain differences in this reproductive parameter.

As for our findings on oestral cycle length, it diverged from other Neotropical felids (e.g., *Leopardus* spp. Moreira *et al*., 2001; *Puma concolor*) or other *Panthera* species (*P. pardus*, Santiapillai *et al*., 1982; Schmidt *et al*., 1988; Schmidt *et al*., 1993; Graham *et al*., 1995)*.* However, when compared to cats of similar size, the duration falls within the same range (*P. tigris*, Brown, 2011; *Puma concolor*, Eisenberg and Redford, 1999; *Pa. leo*, Schmidt *et al.*, 1979; Graham *et al*., 1995; *Pa. uncia*Graham *et al*., 1995). Although differences can be detected visually, our statistical analysis did not confirm such a trend.

Hence, while the phylogenetic signal (i.e. the effect of genus) exists, all size categories explained the variation observed in AgM. Interaction among genus, size, ECL and AgM could not be tested because of small sample size, though it would be interesting to assess this interaction further. Allometric and phylogenetic influence on reproduction is widespread among animals (e.g. Harvey and Zammuto, 1985).

### Concluding remarks

It is necessary to emphasize that the *Panthera* genus is the most diverse Felidae genus and the most well-studied. However, many Felid species remain poorly known regarding its reproductive endocrinology, particularly those of smaller-sized and Asian species in order to understand the patterns in wild cats. Additionally, to collect and preserve female gametes is challenging, and a strong knowledge on physiology is necessary to be successful (Asa, 2012). Therefore, by adding to the body literature on hormonal assessment of wild felids, we can provide a starting point to sister-species. Many species rely on assisted reproduction to maintain viable captive and wild population and it is only possible by induction of oestrus and ovulation (Asa *et al*., 2006). These parameters are of prime importance for wild population conservation and captive welfare.

## Conclusion

Our study is only the second study addressing naturally occurring oestral cycles in female jaguars and, to our knowledge, the first to assess puberty. Our findings agreed with literature that employed hormonal analysis but differed from those based solely on behavioural observations. Such difference could be related to the lack of oestrous behaviour at the beginning of the ovarian activity. Therefore, we emphasize that hormonal assessment is of prime importance for both the onset of ovarian activity but also as a follow-up on reproductive status of adult females. When compared to other felids, our data resembles other *Panthera* representatives if of similar size. An adequate functioning of ovarian hormones is of prime interest for assisted reproduction, occasionally a necessary step for endangered species.

## Funding

This research was supported by Fundação de Amparo à Pesquisa do Estado de São Paulo [process numbers 00/14352-6 and 316/2003].
